# A fast-responsive fluorescent turn-on probe for nitroreductase imaging in living cells†

**DOI:** 10.1039/d0ra09512e

**Published:** 2021-02-24

**Authors:** Chengli Jia, Yong Zhang, Yuesong Wang, Min Ji

**Affiliations:** School of Biological Sciences and Medical Engineering, Southeast University Nanjing Jiangsu 210009 PR China jimin@seu.edu.cn 838826418@qq.com +86-13851570005 +86-13851570005

## Abstract

Nitroreductase (NTR) may be more active under the environment of hypoxic conditions, which are the distinctive features of the multiphase solid tumor. It is of great significance to effectively detect and monitor NTR in the living cells for the diagnosis of hypoxia in a tumor. Here, we synthesized a novel turn-on fluorescent probe NTR-NO_2_ based on a fused four-ring quinoxaline skeleton for NTR detection. The highly efficient probe can be easily synthesized. The probe NTR-NO_2_ showed satisfactory sensitivity and selectivity to NTR. Upon incubation with NTR, NTR-NO_2_ could successively undergo a nitro reduction reaction and then generate NTR-NH_2_ along with significant fluorescence enhancement (30 folds). Moreover, the fluorescent dye NTR-NH_2_ exhibits a large Stokes shift (Δ*λ* = 111 nm) due to the intramolecular charge transfer (ICT) process. As a result, NTR-NO_2_ displayed a wide linear range (0–4.5 μg mL^−1^) and low detection limit (LOD = 58 ng mL^−1^) after responding to NTR. In addition, this probe was adopted for the detection of endogenous NTR within hypoxic HeLa cells.

## Abstract 2

Probe NTR-NO_2_ was effectively reduced in the presence of NTR generating a highly fluorescent product.

## Graphical abstract

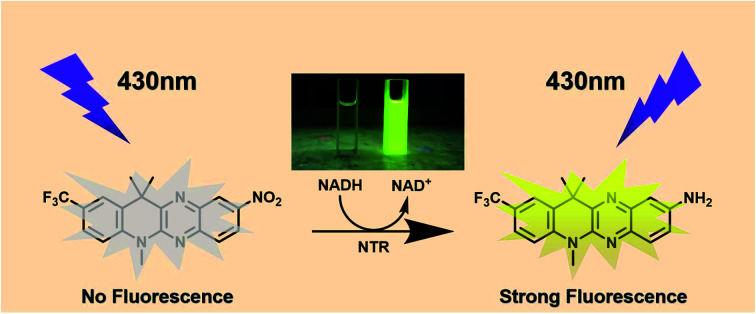

## Introduction

It is found that the average oxygen concentration is in the range of 4% to 0% in the microenvironment of solid tumors.^1^ Hypoxia in tumor growth is caused by the infinite growth of tumor cells, which makes blood supply insufficient and causes vascular dysplasia.^2–5^ Clinical studies found that the tumor hypoxic status is tightly associated with tumor diagnosis and therapy, which exerts a vital part in the metastasis, invasion, as well as drug resistance of the tumor.^6,7^ NTR is a flavinase with nicotinamide adenine dinucleotide (NADH) as a coenzyme, which can catalyze the reduction of nitroaromatic compounds to the corresponding amino compounds.^8–10^ It has been confirmed that hypoxia can result in the overexpression of reductase, such as nitroreductase, DT-diaphorase, and azo reductase.^11–15^ The activity of NTR can be used as an important biomarker to reflect the hypoxic conditions in tumor cells and tissues.^16,17^

Traditional hypoxia detection methods include magnetic resonance imaging, *p*_O_2__ electrodes and positron emission tomography/computed tomography (PET/CT), and it generally requires expensive equipment and complex sample pretreatment.^18–25^ Moreover, the integrity of samples and achieving higher spatial and temporal resolution cannot be guaranteed. Furthermore, fluorescence imaging has attracted widespread attention to monitoring biological molecules owing to its real-time imaging, high sensitivity and selectivity, and non-invasiveness. Numerous fluorescent probes have been designed according to the reduction reaction catalyzed by NTR to detect the hypoxia status for over the last 10 years (Table S1†).^26–33^ However, many fluorescence NTR probes exhibit short Stokes shift (<100 nm) and slight signal changes after responding to NTR, which is easily disturbed by the background fluorescent signal. Thus, there is an urgent requirement to develop simple fluorescent probes with obvious signal changes after responding to NTR.

**Scheme 1:**
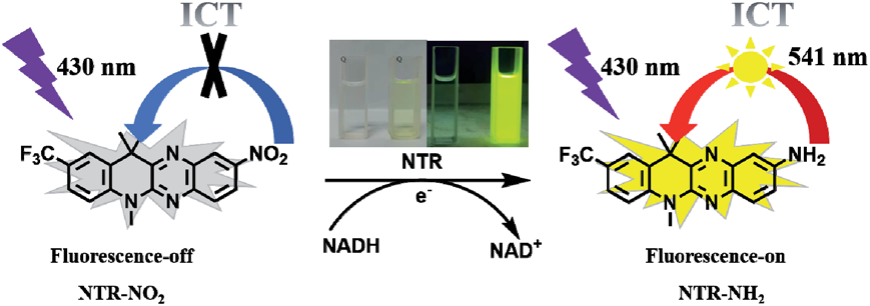
The proposed detection principle of probe NTR-NO_2_ towards NTR.

Here, we successfully synthesized a turn-on fluorescent probe NTR-NO_2_ based on a fused four-ring quinoxaline skeleton reported by our previous work for NTR detection (Scheme 1).^34^ By introducing nitro as the response site, the probe NTR-NO_2_ displayed weak emission due to the well-known quenching effect of a nitro group. The probe shows obvious fluorescence turn-on signal (30-fold) after the nitro to amine reduction reaction catalyzed by NTR with the aid of NADH as an electron source. Furthermore, the activated version of the probe NTR-NH_2_ possessed strong fluorescent emission and large Stokes shift (Δ*λ* = 111 nm) due to ICT it showed low toxicity to cells and high photo-stability, indicating that NTR-NH_2_ is potentially employable as a fluorophore in the probe.^35^ The probe NTR-NO_2_ exhibited fast response and high selectivity to NTR. Good linearity between the fluorescent intensity and the concentration of NTR ranging from 0 to 4.5 μg mL^−1^ was observed. The limit of detection (LOD) was calculated to be 58 ng mL^−1^. Moreover, the probe NTR-NO_2_ was successfully applied in the monitoring of NTR activity within the HeLa cell under hypoxic conditions.

## Experimental

### Materials and apparatus

All chemicals were purchased through chemical reagent corporations and used without further purification. Amino acids and chemical reagents were provided by Annaiji Chemical. Nicotinamide adenine dinucleotide (NADH) and *Escherichia coli* NTR were provided by Solarbio. MTT (3-(4,5-dimethylthiazol-2-yl)-2,5-diphenyl-tetrazolium bromide) was provided by KeyGen Biotech (Nanjing, China). MEM medium and sterile phosphate-buffered saline (PBS) were provided by KeyGen Biotech (Nanjing, China).

The ^1^H NMR and ^13^C NMR spectra were measured on the Qone-WNMR-I-AS400 instrument with DMSO-d6 as the solvent. The Agilent 1100 LC/DAD/MSD was used for high-resolution mass spectrometry (HRMS). Absorption spectra were recorded using a UV3600 UV-VIS-NIR spectrometer. Fluorescence emission spectra were recorded using a FluoroMax-4 fluorescence spectrophotometer. The pH values were measured using the Ray Magnetic pHS-3E pH meter. In addition, FV1000 confocal microscopy was used to perform cell fluorescence imaging.

### Spectroscopic characteristics and NTR optical response

Unless specially stated, the absorption and fluorescence spectra of NTR-NO_2_ (10 μM) at different concentrations of NTR in PBS solution (20% DMSO, pH = 7.4) were recorded at 37 °C in the presence or absence of NADH (500 μM) for 30 min. For all fluorescence spectra measurements, the excitation wavelength was 430 nm, and the slit widths were 2 nm/2 nm.

### Synthesis of NTR-NO_2_

Compound 1 was synthesized following a previously reported method.^36–38^ 4-(Trifluoromethyl)phenylhydrazine hydrochloride (638 mg, 3 mmol) and 3-methyl-2-butanone (387 mg, 4.5 mmol) were dissolved in 5 mL acetic acid while stirring. Then, the resulting mixture was heated to 90 °C and stirred for 12 h. After the reaction was complete, the hot solution was cooled to room temperature. Ethyl acetate was used for the extraction of products from the mixture (3 times). The organic layers were extracted in a vacuum to afford the crude product, which was directly used in subsequent reactions.

Compound 2 was synthesized by a previously reported method.^37,38^ Compound 1 and iodomethane (850 mg, 6 mmol) were dissolved in THF (10 mL). The solution was refluxed at 70 °C for 12 h and a large amount of insoluble solid precipitated from the solution was obtained. Then, the reaction mixture was filtered and the insoluble solid was washed with cold THF. After drying under vacuum, light yellow solid was obtained with a 55% yield.

NTR-NO_2_ was synthesized following a previously reported method.^34^ Compound 2, 2,4-nitrobenzene-1,2-diamine (850 mg, 6 mmol), and iodine (1 g, 4.5 mmol) were dissolved in DMSO (5 mL). Then, the resulting mixture was heated to 100 °C and stirred for 1 h. After the reaction was complete, the mixture was cooled to room temperature and added to saturated Na_2_S_2_O_3_ solution. Then, the mixture was extracted with ethyl acetate (3 times) and evaporated in vacuum. The residue was purified using column chromatography on silica gel with petroleum ether : ethyl acetate (400 : 1, v/v) as the eluent to obtain the products. NTR-NO_2_ (yield 85%): yellow solid; mp 190–191 °C; ^1^H̲ NMR (400 MHz, CDCl_3_) (Fig. S1†): *δ* = 8.90 (d, *J* = 2.3 Hz, 1H), 8.43 (dd, *J* = 9.1, 2.4 Hz, 1H), 7.92 (d, *J* = 9.1 Hz, 1H), 7.78 (s, 1H), 7.64 (d, *J* = 8.4 Hz, 1H), 7.27 (d, *J* = 8.6 Hz, 1H), 3.85 (s, 3H), 1.81 (s, 6H); ^13^C̲ NMR (100 MHz, CDCl_3_) (Fig. S2†): *δ* = 152.6, 146.7, 144.8, 144.4, 140.8, 137.5, 132.2, 127.8, 125.2 (q, *J*_C–F_ = 33 Hz), 125.1 (d, *J*_C–F_ = 4.0 Hz), 125.0, 124.3 (q, *J*_C–F_ = 270 Hz), 123.4, 122.9 (d, *J*_C–F_ = 4.0 Hz), 114.1, 41.1, 31.4, 28.7; HRMS (ESI^+^) (Fig. S3†) calcd for C_19_H_15_F_3_N_4_O_2_ [M + H]^+^ 389.12199, found 389.12210.

### Cytotoxicity assays and hypoxic imaging

HeLa cells were purchased from KeyGen Biotech (Nanjing, China). The cells were cultivated within MEM medium supplemented with 10% fetal bovine serum (FBS), 0.08 mg mL^−1^ streptomycin and 80 U mL^−1^ penicillin and incubated at 37 °C containing 5% CO_2_/95% O_2_ gas. The HeLa cell line was then inoculated in the 96-well plates at 5 × 10^4^ per well. Thereafter, the cultures were removed and the NTR-NO_2_ probe at different contents (0–50 μM) was added to incubate with the cells for 24 h. After adding MTT (20 μL, 5 g L^−1^), cells were subjected to another 4 h of incubation. Later, 100 μL of DMSO was added to replace the original medium and to dissolve formazan crystals. Then, the multifunctional microplate reader was used to measure absorbance (OD) value at 570 nm, and cell viability was presented as follows:^39^
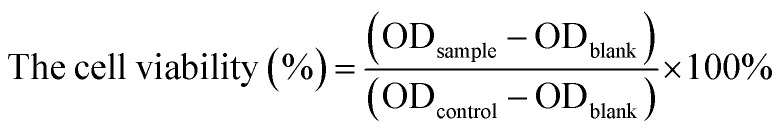


HeLa cells (100 μL) were inoculated onto glass coverslips for adherence. Then, cells were further incubated under 20% O_2_ (normoxia) and 1% O_2_ (hypoxia) for 12 h, followed by further incubation with the probe NTR-NO_2_ for another 30 min under respective conditions. After washing with PBS (pH = 7.4) three times, cells were imaged using confocal fluorescence on a Olympus FV1000 confocal fluorescence microscope.

## Results and discussion

### Synthesis and sensing mechanism of probe NTR-NO2

**Scheme 2:**
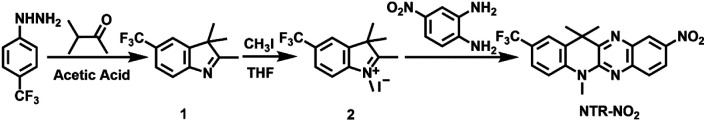
Structure and synthesis of NTR-NO_2_.

**Fig. 1:**
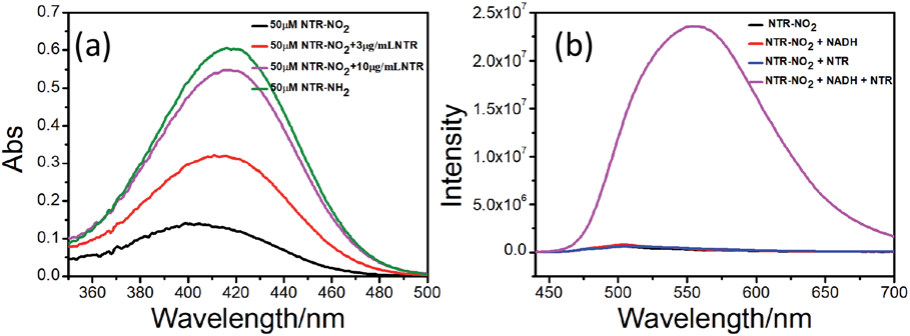
(a) Absorption spectra changes of probe NTR-NO_2_ (50 μM) with or without NTR (0 μg mL^−1^, 8 μg mL^−1^, 10 μg mL^−1^) and the fluorophore NTR-NH_2_ within PBS buffer (pH = 7.4, 20% DMSO) for 20 min. (b) Emission spectra of probe NTR-NO_2_ (10 μM) with or without NTR within PBS buffer (pH = 7.4, 20% DMSO) for 20 min.

We synthesized NTR-NO_2_ as a novel off-on fluorescent probe for NTR detection based on a fused four-ring quinoxaline skeleton reported in our previous work.^34^ The –CF_3_ and –NO_2_ groups were attached to the dihydroquinoline unit and quinoxaline unit in this skeleton, respectively, where the nitro group was selected as the recognition site for NTR and the fluorescence quenching group due to its strong electron attraction. NTR-NO_2_ was synthesized as shown in Scheme 2. The chemical structure of NTR-NO_2_ was identified using NMR and HRMS (Fig. S1–S3†). In this probe, fused cyclic derivatives of quinoxaline structure were employed as e-withdrawing parts for fluorescent push–pull systems due to its highly π-deficient feature;^34^ the probe NTR-NO_2_ exhibited weak emission due to the well-known quenching effect of a nitro group. Subsequently, the nitro group was reduced into the corresponding amino group after the reaction was catalyzed by NTR in the presence of NADH, and it resulted in the formation of the “push–pull” structure of NTR-NH_2_. NTR-NH_2_ was highly emissive upon photoexcitation due to the ICT process reported by our group.^35^ As a result, the obvious turn-on signal was measured. For verifying the above mechanism (Scheme 1), HRMS analysis (Fig. S4†) along with absorption was performed to test the reaction solution of the probe NTR-NO_2_ with NTR (Fig. 1a). As suggested by HRMS for the reaction solution, the main peak was observed at *m*/*z* = 359.1479 [M + H]^+^, which was characterized as NTR-NH_2_. Changes in spectral characteristics of NTR-NO_2_ solution, when NTR was added in the presence of NADH showed that the reaction solution had a similar maximum absorption wavelength to that of NTR-NH_2_. All these results clearly demonstrate that the reaction of NTR-NO_2_ with NTR in the presence of NADH causes the reduction of the nitro group.

### Photophysical performances of the NTR-NO2 probe

**Fig. 2:**
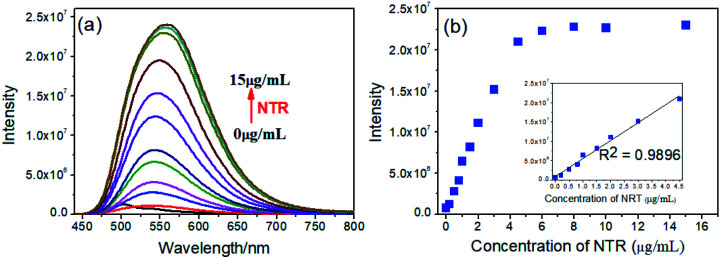
(a) Fluorescence spectra of NTR-NO_2_ (10 μM) at different concentrations of NTR (0, 0.2, 0.5, 0.8, 1, 2, 3, 4.5, 6, 8, 10, 15 μg mL^−1^) in the PBS (pH = 7.4, 20% DMSO) solution for 20 min; (b) the fluorescence intensity of NTR-NO_2_ (10 μM) at 541 nm in the light of NTR concentration; inset: the linear relationship between *I*_541 nm_ and the concentration of NTR.

We investigated optical properties of the NTR-NO_2_ probe with or without NTR at 37 °C using the PBS buffer (pH = 7.4, 20% DMSO) in the presence of 500 μM of NADH. The probe showed a sensitive spectral response toward NTR. It can be seen from Fig. 1a that the free-probe (50 μM) showed a maximum absorption peak around 400 nm. After the addition of NTR in the presence of 500 μM NADH, a new maximum absorption peak at around 425 nm was observed with the chromogenic changes easily detected by the naked eye from the initial colorless to yellow. As shown in Fig. 1b, the free-probe NTR-NO_2_ was nearly non-fluorescence upon excitation at 420 nm, which was in good agreement with the absorption spectra. Upon excitation at 430 nm, it was obvious that the fluorescence intensity at 541 nm showed gradual enhancement upon the NTR titration in the presence of NADH (Fig. 2).

**Fig. 3:**
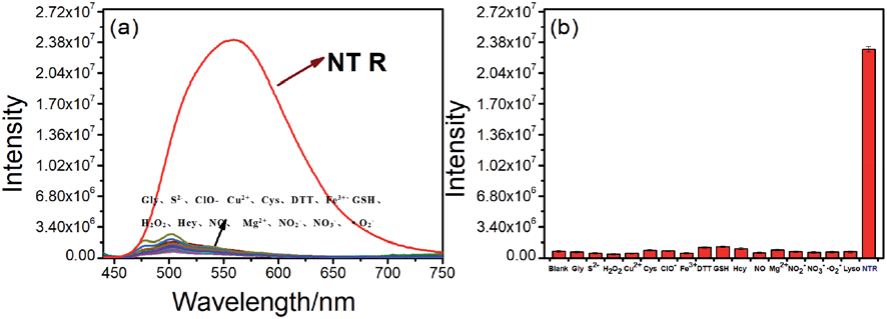
Fluorescence responses of NTR-NO_2_ (10 μM) to various species (500 μM) in the presence of NADH (500 μM) in the PBS buffer (pH = 7.4, 20% DMSO) for 20 min: (0) Blank, (1) Fe^3+^, (2) Mg^2+^, (3) H_2_O_2_, (4) Ca^2+^, (5) Gly, (6) ClO^−^, (7) NO_2_^−^, (8) DTT, (9) CYS, (10) GSH, (11) NO_3_^−^, (12) ·O_2_^−^, (13) S^2−^, (14) NO, (15) HCY, (16) Fe^2+^ and (17) NTR (8 μg mL^−1^).

To demonstrate the sensitivity and selectivity of NTR-NO_2_, the fluorescence spectra of NTR-NO_2_ (10 μM) with NTR in various concentrations in the presence of 500 μM NADH were measured in the PBS buffer (pH = 7.4, 20% DMSO). Importantly, the emission intensity of NTR-NO_2_ (10 μM) at 541 nm was enhanced approximately 30-folds after incubating with NTR (6 μg mL^−1^) in the presence of 500 μM NADH. The plots of *I*_541 nm_ (*λ*_ex_ = 420 nm) against the concentrations of NTR ranging from 0 μg mL^−1^ to 4.5 μg mL^−1^ exhibited a good linear relationship (0.9896) (Fig. 2). Moreover, the detection limit of NTR-NO_2_ toward NTR was determined to be 58 ng mL^−1^. Compared with the probe NTR-NO_2_, the fluorescence quantum yield (*Y*_s_) of the active version after responding to NTR increased by nearly 23-folds (from 0.019 to 0.43) (Table S2†). In addition, the probe NTR-NO_2_ (10 μM) was incubated with various biologically relevant species (500 μM) in the presence of 500 μM NADH to assess the selectivity of NTR-NO_2_ towards NTR. As shown in Fig. 3, the probe NTR-NO_2_ showed apparent fluorescence changes only in the presence of NTR. Other biologically relevant substances cannot cause significant fluorescence changes even at higher concentrations. Moreover, we further incubated the probe of NTR-NO_2_ with a strong reducing agent NaBH_4_ in the presence of NADH. No notable fluorescence changes were observed (Fig. S5†). These results revealed that the probe NTR-NO_2_ could be highly-sensitive and highly selective to monitor the level of NTR by the remarkable turn-on signal.

### Time-dependence and pH effect of NTR-NO2 to NTR

**Fig. 4:**
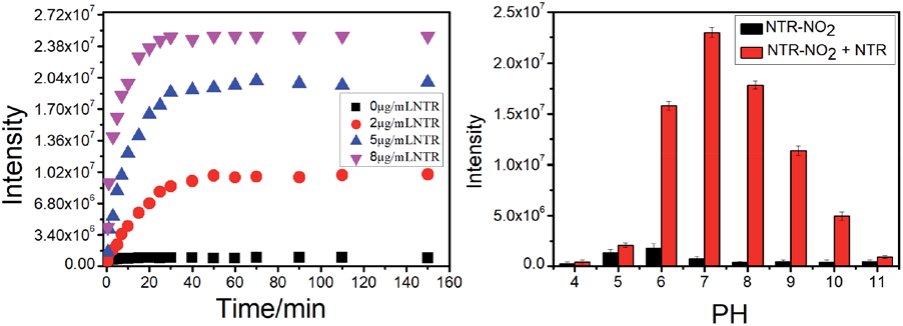
(a) The plot of fluorescence intensity at 541 nm of NTR-NO_2_ (10 μM) with respect reaction time for varied concentrations of NTR in the presence of 500 μM NADH at 37 °C. (b) Effect of pH on the fluorescence intensity changes of probe NTR-NO_2_ (10 μM) at 541 nm in absence (black) and presence (red) of NTR (8 μg mL^−1^) in PBS buffer (20% DMSO) for 20 min.

The kinetic profiles of the probe NTR-NO_2_ (10 μM) incubated with different levels of NTR (0–8 μg mL^−1^, 500 μM NADH) was studied. As shown in Fig. 4a, the solution of the probe NTR-NO_2_ gradually displayed a fluorescence increase at 541 nm upon the addition of NTR in the presence of NADH, which was saturated over 20 min. Moreover, no fluorescence intensity change was observed in the free-probe, indicating that the probe was stable in such a buffer system. According to the above kinetic results, the probe NTR-NO_2_ may be used to rapidly detect NTR.

Furthermore, the pH effect on the detection of NTR activity was investigated to assess the potential applications of the probe NTR-NO_2_ under different conditions. The probe NTR-NO_2_ (10 μM) showed weak emission intensity in diverse pH environments, and the fluorescence intensity increased significantly after responding to NTR (8 μg mL^−1^) in the presence of NADH within a wide pH range (Fig. 4b). It suggested that our probe could be applied to rapidly monitor NTR in the pathological or physiological condition.

### Fluorescence imaging of NTR within living cells

**Fig. 5:**
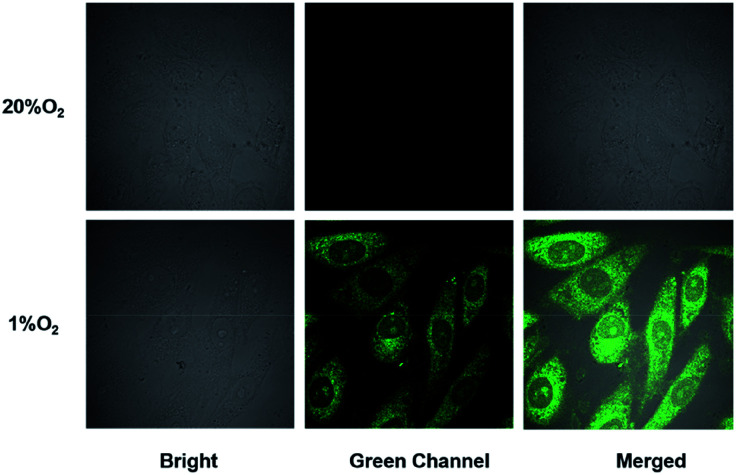
Fluorescence imaging of NTR in the living HeLa cells under normoxic (20% *p*_O_2__) and hypoxic (1% *p*_O_2__) conditions incubated with the probe NTR-NO_2_ for 30 min under 37 °C.

Encouraged by the favorable properties of the probe NTR-NO_2_, we conducted the fluorescence imaging experiments in living cells. First, we evaluated the cytotoxicity of the probe NTR-NO_2_*via* MTT assays. As shown in Fig. S6,† it indicated that the probe NTR-NO_2_ has no remarkable cytotoxicity to the cells even up to 50 μM. Then we assessed whether it could monitor intracellular hypoxia. HeLa cells were incubated with probe NTR-NO_2_ (10 μM) under normoxic (20% *p*_O2_) and hypoxic (1% *p*_O2_) conditions. As shown in these confocal fluorescence images (Fig. 5), there was only weak fluorescence emission under 20% *p*_O2_ condition. However, an obvious fluorescence intensity could be observed under 1% *p*_O2_ condition resulted in more active NTR and in the formation of NTR-NH_2_. The above findings demonstrated that our probe could be used to monitor endogenous NTR in tumor cells.

## Conclusion

In summary, a novel turn-on probe NTR-NO_2_ was prepared in this study based on the fused four-ring quinoxaline skeleton detecting NTR. This probe exhibited significant fluorescence emission change catalyzed by NTR in the presence of NADH based on ICT. Our probe showed high sensitivity and selectivity toward NTR. Moreover, we have demonstrated that the probe NTR-NO_2_ can be effectively used to monitor hypoxia *via* the detection of NTR in HeLa cells. We anticipate that the probe NTR-NO_2_ could be used as a powerful tool for investigating the biological functions of NTR *in vivo*.

## Conflicts of interest

There are no conflicts to declare.

## Supplementary Material

RA-011-D0RA09512E-s001
