# Supplementary material for: A fast-responsive fluorescent turn-on probe for nitroreductase imaging in living cells
Source: RSC Adv. 2021 Feb 24;11(15):8516–20. doi: 10.1039/d0ra09512e (PMC8695130; doi:10.1039/d0ra09512e)
Supplement: RA-011-D0RA09512E-s001 [file RA-011-D0RA09512E-s001.pdf]

Electronic Supplementary Material (ESI) for RSC Advances.
This journal is © The Royal Society of Chemistry 2021

Supporting Information

A fast-responsive fluorescent turn-on probe for nitroreductase imaging in living cells

Chengli Jia, Yong Zhang, Yuesong Wang, Min Ji*

School of Biological Sciences and Medical Engineering, Southeast University, Nanjing, 210009

Contents

1. Reported fluorescent probes....2

2. The characterization of NTR-NO_2....3

3. The measurement of fluorescence quantum yields....4

4. The HRMS analysis of the products....5

5. The fluorescent spectra of NTR-NO_2 responding with $NaBH_4$....6

6. Cytotoxicity assays of probe NTR-NO_2 at different concentrations....6

7. Reference....7

1. Reported fluorescent probes

Table S1. Comparison of fluorescent probes for palladium detection

Probe	$\lambda_{ex}/\lambda_{em}$ (nm)	Stokes shift (nm)	Response time (min)	Limit of detection (ng/mL)	Reference
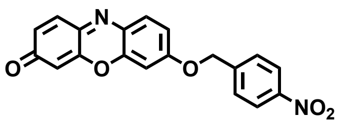	564/586	22	90	————	*Dyes and Pigments* **2019,** *171.*
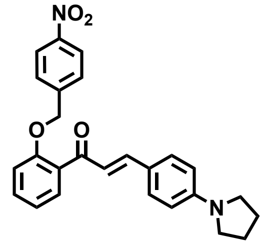	467/526	59	60	27	*Sensors and Actuators B: Chemical* **2018,** *276,* 397-403.
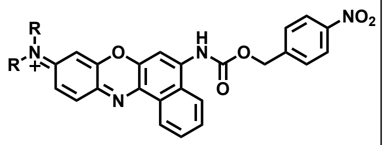	613/658	45	70	180	*Chem. Commun.,* ***2013****, 49, 10820–* [illegible]
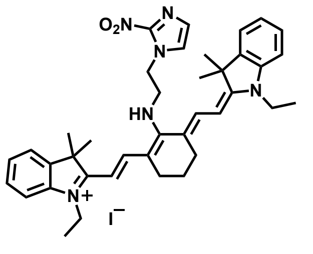	695/750	55	15	77	*Chem. Commun.,* ***2013****, 49, 2554–2556.*
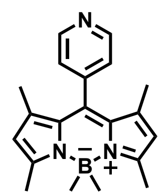	470/520	50	5	9.6	*Analyst,* ***2015****, 140,*
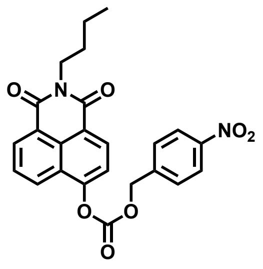	450/550	100	30	————	*J. Photochem. Photobiol. A Chem.* ***2018****，353， 292–* [illegible]

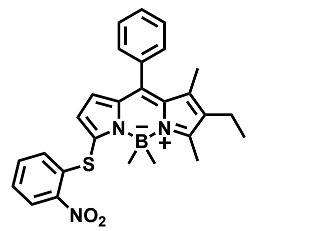	450/540	90	180	22	*Organic & Biomolecular Chemistry* **2020,** *18* (25), 4744-4747
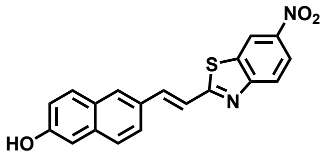	405/534	129	60	48	*Analyst* **2020,** *145* (16), 5657-5663
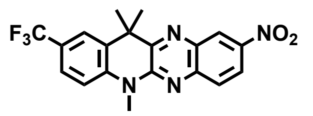	430/541	111	20	58	**This work**

2. The characterization of NTR-NO_2

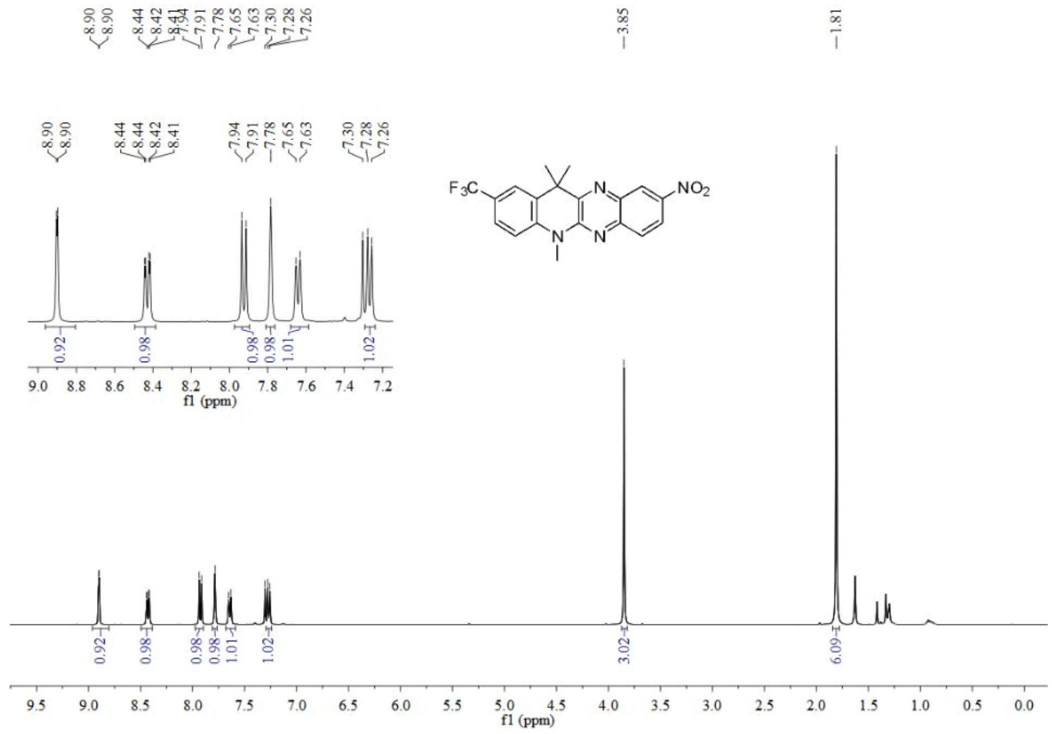

Fig. S1: ^{1}H NMR spectrum of **NTR-NO_2**

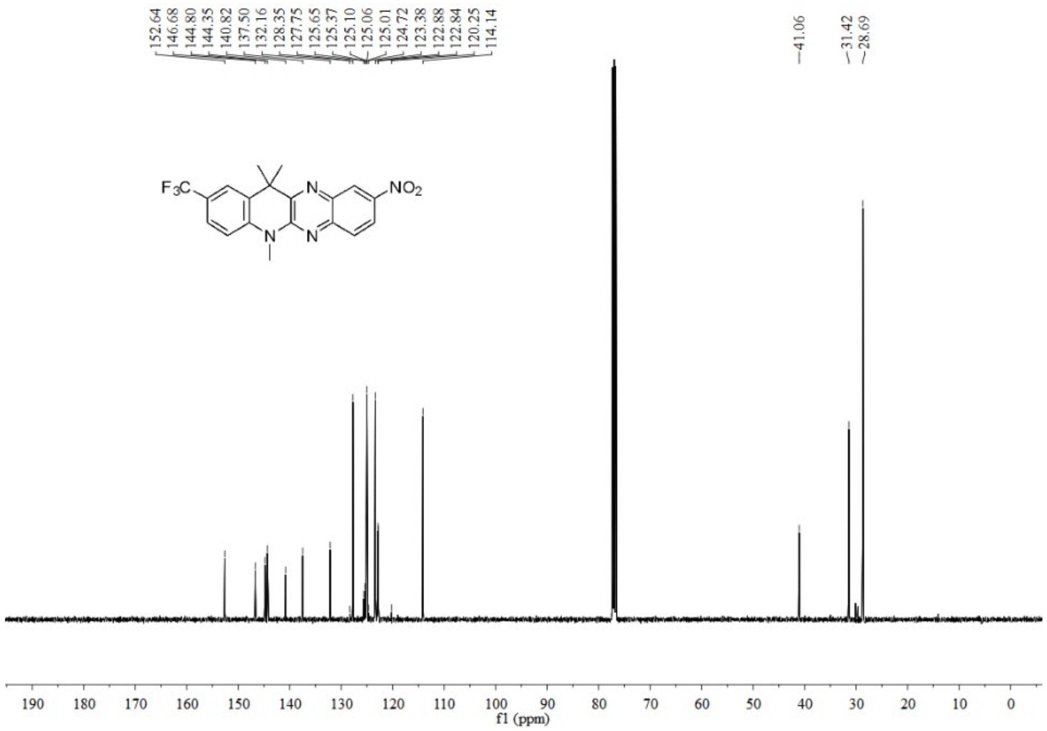

Fig. S2: ^{13}C NMR spectrum of **NTR-NO$_2$**

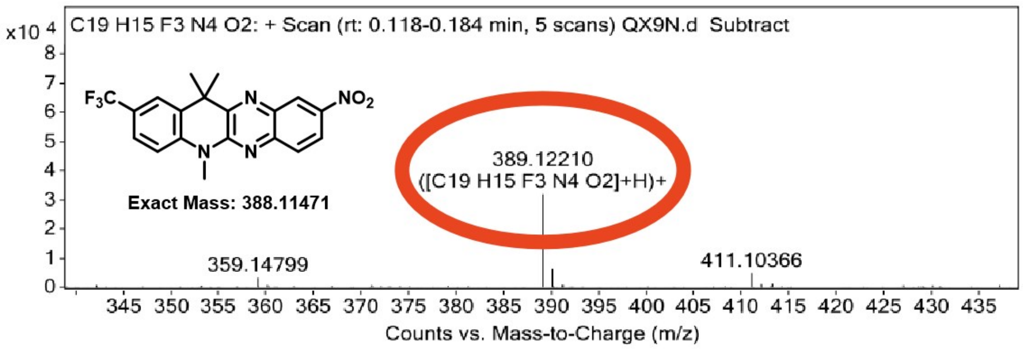

Fig. S3: HRMS spectrum of **NTR-NO$_2$**

3. The measurement of fluorescence quantum yields

The quantum yield values were calculated by using coumarin-153 in ethanol (Φ = 0.38) as a standard according to the following formula[1-3] :

$$Y_u = Ys \bullet \frac{F_u}{F_s} \bullet \frac{A_s}{A_u} \bullet \left[\frac{G_u}{G_S} \right]^2$$

Where, Y_u is the quantum yield of NTR-NH_2; Y_s is the quantum yield of coumarin-153 (Φ= 0.38) in ethanol; F is the integrated emission intensity (peak area); A is the absorbance at λ_{ex};

Table S2. Photophysical properties of **NTR-NH$_2$**

(DMSO:PBS=1:5, pH = 7.4)

Compound	λ_{abs} (nm)	λ_{em} (nm)	Stokes shift (nm)	Y_u
**NTR-NH$_2$**	430	541	111	0.43

4. The HRMS analysis of the products

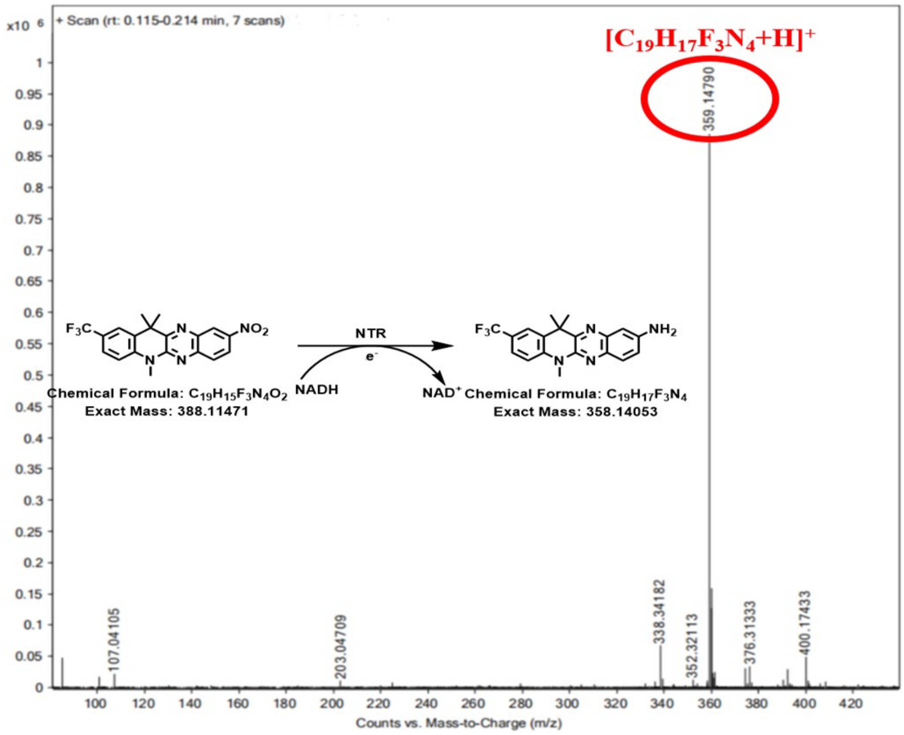

Fig. S4: HRMS spectrum of **NTR-NO$_2$**

5. The fluorescent spectra of $NTR-NO_2$ responding with $NaBH_4$

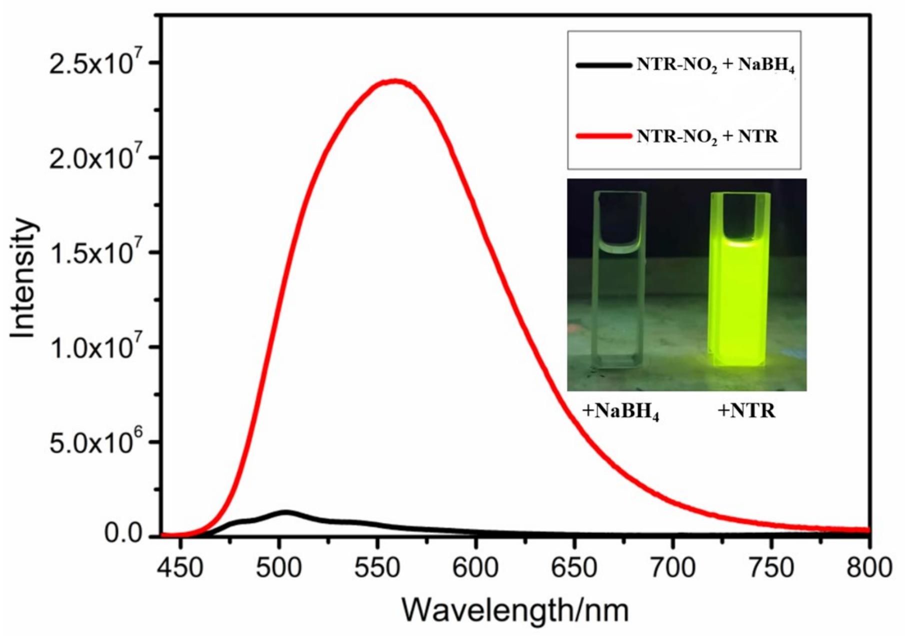

Fig. S5: The fluorescence spectra of probe **$NTR-NO_2$** (10μM) incubated with NTR (red) and $NaBH_4$ (black) in the presence of NADH (500μM)

6. Cytotoxicity assays of probe $NTR-NO_2$ at different concentrations

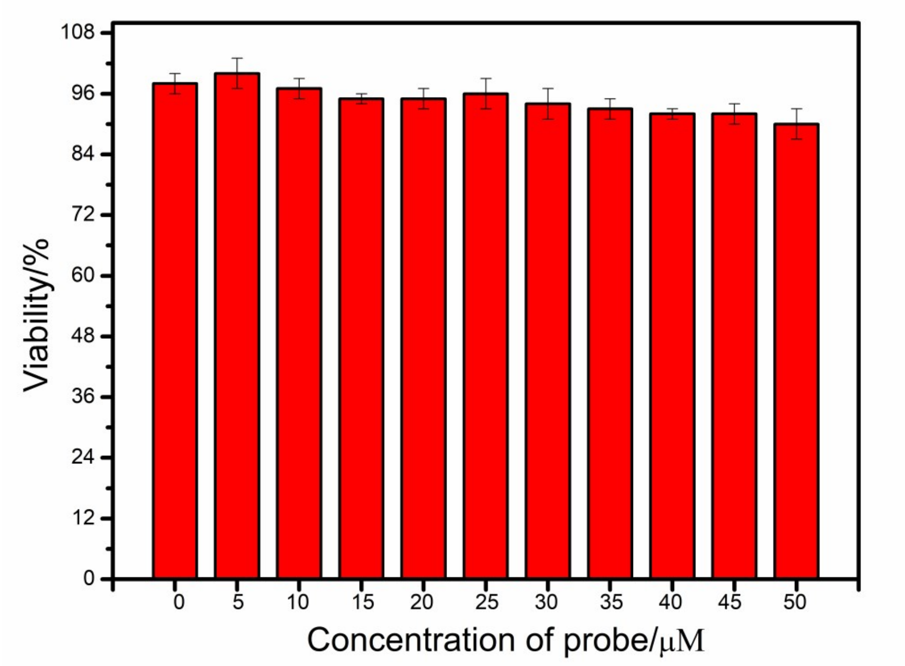

Fig. S6: MTT assay for the viability of HeLa cells treated with various concentrations of probe **$NTR-NO_2$** for 24h

7. Reference

1. D. Guo, Z. P. Dong, C. Luo, W.Y. Zan, S. Q. Yan and X. J. Yao, RSC Adv., 2014, 4, 5718-5725.
2. C. Kar, M. A. Adhikari, A. Ramesh and G. Das, Inorg. Chem., 2013, 52, 743-752.
3. D. R. Haynes, A. Tokmakoff, S. M. George, Chemical Physics Letters, 1993, 214, 50-56.
